# The Role of Edge-Based and Surface-Based Information in Incidental Category Learning: Evidence From Behavior and Event-Related Potentials

**DOI:** 10.3389/fnint.2020.00036

**Published:** 2020-07-22

**Authors:** Xiaoyan Zhou, Qiufang Fu, Michael Rose

**Affiliations:** ^1^State Key Laboratory of Brain and Cognitive Science, Institute of Psychology, Chinese Academy of Sciences, Beijing, China; ^2^Department of Psychology, University of Chinese Academy of Sciences, Beijing, China; ^3^The Research Center for Psychological Education, University of International Relations, Beijing, China; ^4^NeuroImage Nord, Department for Systems Neuroscience, University Medical Center Hamburg Eppendorf, Hamburg, Germany

**Keywords:** edge-based information, surface-based information, cross-modal category learning, incidental category learning, event-related potentials

## Abstract

Although it has been demonstrated that edge-based information is more important than surface-based information in incidental category learning, it remains unclear how the two types of information play different roles in incidental category learning. To address this issue, the present study combined behavioral and event-related potential (ERP) techniques in an incidental category learning task in which the categories were defined by either edge- or surface-based features. The results from Experiment 1 showed that participants could simultaneously learn both edge- and surface-based information in incidental category learning, and importantly, there was a larger learning effect for the edge-based category than for the surface-based category. The behavioral results from Experiment 2 replicated those from Experiment 1, and the ERP results further revealed that the stimuli from the edge-based category elicited larger anterior and posterior P2 components than those from the surface-based category, whereas the stimuli from the surface-based category elicited larger anterior N1 and P3 components than those from the edge-based category. Taken together, the results suggest that, although surface-based information might attract more attention during feature detection, edge-based information plays more important roles in evaluating the relevance of information in making a decision in categorization.

## Introduction

A fundamental question in category learning is how the category knowledge is extracted and represented in the human brain. The prototype theory posits that people form a summary representation in the form of prototypes in category learning (Knowlton and Squire, 1993; Reber et al., 1998a,b; Reed et al., 1999; Smith and Minda, 2002; Smith, 2002; Bozoki et al., 2006; Homa et al., 2011). The exemplar theory posits that people store categorical members as individuated memory representations in category learning (e.g., Nosofsky and Zaki, 2002; Zaki and Nosofsky, 2004, 2007; Tunney and Fernie, 2012). The rule-based theory, however, contends that people extract verbal rules of prominent features as the category representations in category learning (Maddox et al., 2003; Maddox and Ashby, 2004; Ashby and Maddox, 2005, 2011; Carpenter et al., 2016; Ashby and Valentin, 2017). The above theories differ in the exact content of the category representation; all of them focus on what type of category structure is formed in category learning but ignore the issue of whether the category representation includes primarily edge- or surface-based features.

Edge-based features (e.g., line, shape, and contour) often appear at boundaries to separate an object from its background, whereas surface-based characteristics (e.g., color, brightness, and texture) always define the physical description of a stimulus (Tanaka et al., 2001; Hagen et al., 2014). It has been demonstrated that the representation mediating initial object recognition contains edge-based information such as an object’s shape but not surface-based information such as its color or texture (Biederman, 1987; Biederman and Ju, 1988; Elder and Velisavljević, 2009; Rokszin et al., 2015). It has been also found that surface-based information such as color facilitates recognition only when a stimulus is presented for a relatively long period of time (Laws and Hunter, 2006; Fu et al., 2016) or when objects belong to structurally similar categories with a high color diagnostic (Tanaka and Presnell, 1999; Nagai and Yokosawa, 2003; Bramão et al., 2011, 2012). Importantly, although color photographs include both edge- and surface-based information, while line drawings include only edge-based information, the neural activation in response to line drawings is similar to that for color photographs, indicating that the information included in the line drawings might be equivalent to the original objects or scenes they depict (Sayim and Cavanagh, 2011; Walther et al., 2011; Fu et al., 2016).

If object representation consists primarily of edge-based information, it can be expected that edge-based information might play a more crucial role than surface-based information in category learning, as both include the processing of current stimuli and the comparison between the current stimuli and their internal representations. Indeed, it has been demonstrated that people perform much better when the category is defined by the edge-based features than by the surface-based features, indicating that the two types of information play different roles in category learning (Zhou et al., 2019). However, it remains unclear how the two types of information play different roles in category learning.

Object categorization has been described as a two-stage process (Vanrullen and Thorpe, 2001; Palmeri and Gauthier, 2004; Ungerleider and Bell, 2011; Taminato et al., 2014; Serre, 2016). During the first stage, visual features such as color, motion, and texture are processed, and the proximal representation of the current stimulus is formed in the primary visual cortex and the extrastriata visual cortex (Riesenhuber and Poggio, 2000; DiCarlo et al., 2012). The extraction of visual features is often reflected by early event-related potential (ERP) components including the posterior P1 and N1 and the anterior N1 and P2 prior to about 200-ms poststimulus onset (Freedman et al., 2003; Scholl et al., 2014). The posterior P1 component indexes early sensory processing and is sensitive to attention allocation (Anllo-Vento and Hillyard, 1996; Luck et al., 2000; Fabre-Thorpe et al., 2001; Martínez et al., 2006), whereas the posterior N1 component reflects a discrimination process and also indicates a benefit of exogenous (i.e., bottom–up) attention (Vogel and Luck, 2000; Curran et al., 2002; Chen et al., 2006; Marzecová et al., 2018). The anterior N1 component is observed with a peak latency approximately halfway between the posterior P1 and N1 latencies (Luck and Kappenman, 2012) and reflects the top–down (i.e., voluntary, endogenous) control needed for focusing attention on stimuli (He et al., 2004, 2008; Marzecová et al., 2018). For example, there is a larger anterior N1 component when the cue and the target are presented at the same location than at different locations (He et al., 2004, 2008). In addition, the anterior P2 component has been linked to the detection and analyses of target visual features (Hillyard and Münte, 1984; Luck and Hillyard, 1994; Luck, 2012). For example, there is a larger anterior P2 component for stimuli containing target features compared to stimuli missing several features (Federmeier et al., 2005; Chen et al., 2006; Gratton et al., 2009).

During the second stage, the information of the current stimuli is compared with internal categorical representations to make a decision (Ungerleider and Bell, 2011; Taminato et al., 2014). The evaluation of information relevance in making a decision is more likely to be reflected by relatively late ERP components including the posterior P2, the anterior P3a, and the posterior P3b after about 200 ms of the stimulus onset (Scholl et al., 2014). The posterior P2 might be engaged in more complex encoding processes including the reactivation of stored information and evaluative processes that occur when a visual input is compared with an internal representation (Dunn et al., 1998). It has been found that there is a shorter posterior P2 latency for easily categorizable stimuli (letters or geometrical figures) than hardly categorizable stimuli (structured textures and Asiatic characters; Pernet et al., 2003). The anterior P3a component displays maximum amplitude over frontal/central electrode sites and might reflect a mixture of category selection and categorization uncertainty with enhanced responses to stimuli at the category boundary (Scholl et al., 2014). The P3b components are typically highest on the scalp over parietal brain areas and are related to task demanding and cognitive resources (Polich, 2007). In addition, noncategory members elicit larger posterior P3b components than categorical members (Folstein et al., 2008).

In the current study, to investigate how edge- and surface-based information play different roles in category learning, we adopted behavioral and ERP techniques in an incidental category learning paradigm in which the categories were defined by either edge- or surface-based features. The purpose of Experiment 1 was to explore whether participants could simultaneously acquire the representations of categories defined by edge- and surface-based features and whether edge-based information plays a more important role than surface-based information in incidental category leaning. If edge-based information plays a more primary role than surface-based information in category learning, we would expect that the learning effect would be higher for the category defined by edge-based features than those defined by surface-based features as in Zhou et al. (2019). In Experiment 2, the ERPs technique was used to investigate how the two types of information would play different roles in category learning. If the category representation consists of primarily edge-based information, the categorization based on edge- and surface-based features would differ in early and later ERP components.

## Experiment 1

**Figure 1:**
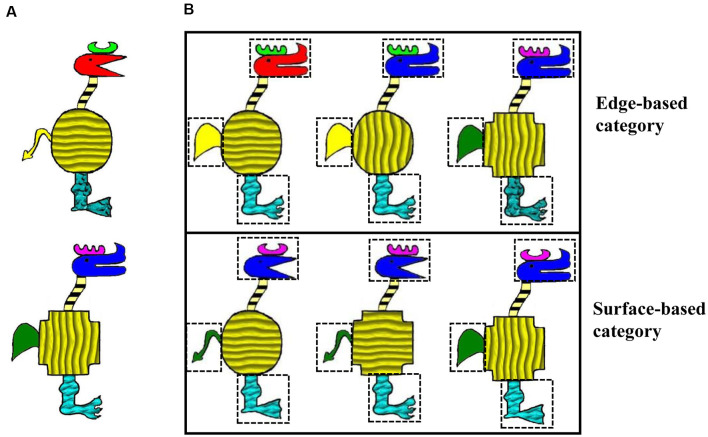
Stimulus examples. **(A)** Two stimulus examples that varied on the features of 10 dimensions. **(B)** Examples of categorical members for each category.

We adapted the stimuli from Gorlick and Maddox (2013) in which cartoon animals were constructed from 10 binary dimensions and each dimension has two features. For example, the shape of the horn can be like a comb or the moon, and the shape of the head can be acutilingual or lamellirostral. To compare the roles of edge- and surfaced-based features in category learning, we maintained five edge-based dimensions including the shapes of the horn, head, body, tail, and leg and added five corresponding surface-based dimensions including the color of the horn, head, tail, and the texture of the body and leg. As a result, the current stimuli varied along 10 binary dimensions, with five edge- and five surface-based dimensions (see Figure 1A). It has been demonstrated that when the category is defined by a four-feature-based rule, participants perform better when the category is defined by edge-based features than by surface-based features (Zhou et al., 2019). Thus, in the present study, the two categories were defined by a four-feature-based rule of either edge- or surface-based features. To investigate whether participants could simultaneously acquire the two categories and express a learning advantage for the category defined by the edge-based features, the stimuli from both categories were presented in the training phase, and unbeknownst to participants, the stimuli of each category were always accompanied by the same type of sound. Participants were asked to rate how likeable the cartoon animal and the sound were on each trial in the training phase.

### Methods

#### Participants

Twenty-five university students (14 female, mean age = 22.16 years, *SD* = 1.95) voluntarily participated in the experiment. They were paid for their attendance. All of them reported normal or corrected to normal vision. The experiment was approved by the Institutional Review Board of the Institute of Psychology, Chinese Academy of Sciences. Data from two participants were excluded from further analysis because their accuracy for both categories was below chance (0.5), and data from one participant were excluded because his accuracy for the surface-based category was above 2 standard deviations from the mean accuracy.

#### Materials

The visual stimuli were cartoon animals that varied along 10 binary dimensions, with five edge-based dimensions including the shape of the horn, head, body, tail, and leg, and five surface-based dimensions including the color of the horn, head, tail, and the texture of the body and leg. Each dimension has two features. Each category was defined by a four-feature-based rule of different types of features. For the edge-based category, the category members were defined by the shape of the horn, tail, leg, and head; correspondingly, for the surface-based category, the category members were defined by the color of the horn and tail, the texture of the leg, and the color of the head (see Figure 1B). Specifically, for the edge-based category, category members were those with a comb horn, a paw-shaped leg, a short and round tail, and a bent head; for the surface-based category, category members were those with a violet horn, a cuspidal leg, a green tail, and a blue head. The features of the four defined dimensions were fixed, while the features of the other six dimensions could change randomly. Thus, there were a total of 64 members in each category. Because four category members could be classified to both categories, they were excluded in the training phase. For each category, 20 category members were presented in the training phase, and the other 40 were presented in the test phase. The four stimuli that belonged to both categories were presented twice in the test phase.

The auditory stimuli were two types of instrument sounds: one was guitar sound, and the other was sand hammer sound. They were produced by the software GarageBand and presented with the same volume (80 db).

#### Procedure

**Figure 2:**
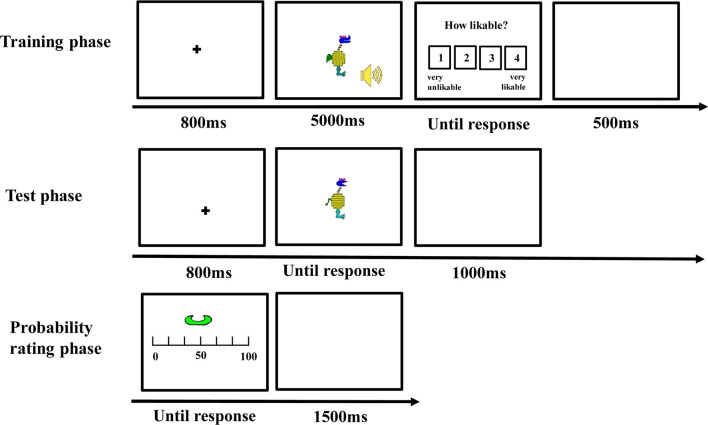
The trial procedure of different phases in Experiment 1.

There was a training phase, a test phase, a probability rating phase, and an importance rating phase (see Figure 2) for each participant.

##### Training Phase

The stimuli were presented on a 17-inch cathode-ray tube (CRT) monitor and subtended a visual angle of <12° (see Nosofsky et al., 2012). Each trial began with a fixation cross at the center for 800 ms, and then, a visual stimulus and a sound were presented for 5,000 ms. Participants were instructed to observe the visual stimulus and listen to the sound carefully during their presentation. After the stimuli disappeared, they were asked to rate how likeable the cartoon animal and the sound were from 1 (very unlikeable) to 4 (very likeable). The intertrial interval was 500 ms. Unbeknownst to the participants, the stimuli of each category were always accompanied by the same type of sound. The combination of the category and the sound was counterbalanced between participants. There were 20 trials for each category, for a total of 40 trials in the training phase. All the trials appeared in a random sequence.

##### Test Phase

After the training phase, participants were informed that the visual stimuli they had rated could be divided into two categories (i.e., “category with guitar” or “category with sand hammer”) according to the sound they were accompanied with during the training phase. Then, they were asked to classify some novel visual stimuli according to the category knowledge they acquired in the training phase. On each trial, a visual stimulus appeared and remained on the screen until participants made classification by pressing one of the two keys with labels “guitar” or “sand hammer” on the keyboard. After the response, the next trial was initiated following a 1,000-ms intertrial interval. There were 88 test trials, of which 40 belonged to the edge-based category, 40 belonged to the surface-based category, and eight belonged to both categories.

##### Probability Rating Phase

During this phase, each defined dimension with different features such as comb-like horn in blue was presented, and participants were asked to report when a stimulus included the features displayed, what was the probability it belonged to the category accompanied with guitar, and the category accompanied with sand hammer separately. Participants were asked to indicate the probability on a continuous sliding scale from 0 to 100, where 0 = definitely no, 50 = equally likely to be yes or no, and 100 = definitely yes. Each defined dimension of the two categories was presented two times, and thus, there were 16 trials in the probability rating phase.

##### Importance Rating Phase

Finally, the names of the 10 dimensions including five edge-based dimensions and five surface-based dimensions were listed in a questionnaire, and participants were asked to rate how important each dimension was when they classified the stimuli on a continuous scale from 0 to 100, where 0 = not important at all, 50 = moderately important, and 100 = very important.

### Results

#### Accuracy in the Test Phase

**Figure 3:**
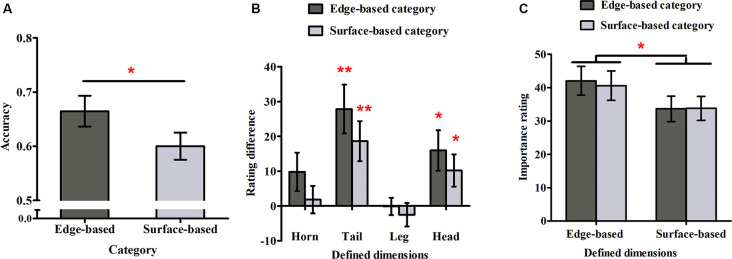
Accuracy and ratings in Experiment 1. **(A)** The accuracy for the edge- and surface-based categories in the test phase. **(B)** The probability rating differences of the defined dimensions between the edge- and surface-based category. **(C)** The importance ratings for the defined dimensions of the edge- and surface-based categories. Error bars depict standard errors. *p < 0.05, **p < 0.01.

The responses for the eight stimuli that belonged to both categories were excluded from this analysis because they could not be divided into correct and incorrect ones. Figure 3A shows the accuracy for each category in Experiment 1. To examine whether participants could simultaneously learn the two categories incidentally, a one-sample *t*-test was used to compare the performance with chance (0.50) for each category. The accuracy for both categories were significantly above chance (edge-based: *M* = 0.69, *SD* = 0.14, *t*_(21)_ = 6.36, *p* < 0.001, Cohen’s *dz* = 1.36; surface-based: *M* = 0.61, *SD* = 0.10, *t*_(21)_ = 5.01, *p* < 0.001, Cohen’s *dz* = 1.07), indicating that participants learned the two categories incidentally at the same time. To explore the role of different features in category learning, we conducted a paired-samples *t-test*, which revealed that the accuracy for the edge-based category was significantly higher than that for the surface-based category [*t*_(21)_ = 2.68, *p* < 0.05, Cohen’s *dz* = 0.57]. Thus, consistent with the previous research (Zhou et al., 2019), the results suggested that participants performed better when the category was defined by edge-based features than by surface-based features.

#### Probability Ratings

To explore whether participants could be aware of the relation between the defined features and the category membership, we first calculated the average rating when the defined dimension had or did not have the defined features separately and then obtained the difference ratings between them (see Figure 3B). If the difference rating was significantly above zero, it would indicate that participants might be aware of the relation between the defined features and the category membership, and *vice versa*. A one-sample *t-test* revealed that for the edge-based category, the difference ratings of the tail and head shapes were significantly above zero (tail shape: *t*_(21)_ = 4.04, *p* < 0.01, Cohen’s *dz* = 0.86; head shape: *t*_(21)_ = 3.08, *p* < 0.05, Cohen’s *dz* = 0.66); for the surface-based category, the difference ratings of the tail and head colors were significantly above zero (tail color: *t*_(21)_ = 3.40, *p* < 0.01, Cohen’s *dz* = 0.72; head color: *t*_(21)_ = 2.37, *p* < 0.05, Cohen’s *dz* = 0.51). The results indicated that participants were partially aware of the relation between the defined features and the category membership.

To explore whether participants could be more aware of the relation between the defined features and the categorical membership for one category than the other one, a 2 (category: edge- vs. surface-based) × 2 (significant defined dimensions: tail vs. head) within-subject ANOVA on the significant difference ratings was conducted. The results revealed that the main effect of defined dimensions was significant (*F*_(1, 21)_ = 2.96, *p* = 0.10) and the interaction effect (*F*_(1, 21)_ = 0.01, *p* = 0.92) were not significant. Importantly, the main effect of category was not significant (*F*_(1, 21)_ = 1.53, *p* = 0.23). Nothing at all follows from a nonsignificant result in itself, but a Bayes factor (*B*) can indicate substantial evidence for the null hypothesis (*B* < 1/3), that the data are insensitive (1/3 < *B* < 3), or substantial evidence for the alternative (*B* > 3; Dienes, 2011, 2014; Fu et al., 2016). Therefore, we calculated the Bayes factor *B* for the difference ratings between the two categories, using the free online calculator on the website from Dienes (2008). The mean difference of the difference ratings between the two categories was 8.52; the standard error of the difference was 6.89. Using the uniform range (0, 100) to represent the alternative (where 100 was the extreme situation when participants acquired completely explicit knowledge for the edge-based category, but they did not acquire any explicit knowledge for the surface-based category, i.e., the difference was 100, while 0 was the extreme situation when participants acquire similar explicit knowledge for the edge- and the surface-based category, i.e., the difference was 0), it yields *B* = 0.32, providing strong evidence that there was no difference in explicit knowledge between the two categories.

#### Importance Ratings

To explore whether participants were more reliant on edge- or surface-based features in classification, we calculated the mean importance ratings for the four edge- or surface-based defined dimensions, when participants classified the stimuli to the two categories separately (see Figure 3C). A 2 (dimensions: edge- vs. surface-based) × 2 (category: edge- vs. surface-based) within-subjects ANOVA revealed only a significant effect of dimensions (*F*_(1, 21)_ = 8.04, *p* < 0.05, $\eta_{\text{p}}^{2}$ = 0.28). The main effect of category (*F*_(1, 21)_ = 0.26, *p* = 0.61) and the interaction (*F*_(1, 21)_ = 0.18, *p* = 0.68) did not reach significance. Similarity, we calculated the Bayes factor *B* for the importance rating difference between the two categories. The mean importance rating difference between the two categories was 1.47, and the standard error was 2.85. Using the uniform range (0, 100) to represent the alternative (where 100 was the extreme situation when the defined dimensions were rated with 100 for the edge-based category but the defined dimensions were rated with 0 for the surface-based category, i.e., the difference was 100, while 0 was the extreme situation when the defined dimensions were rated with similar importance ratings for the edge- and surface-based categories, i.e., the difference was 0), it yields *B* = 0.06, providing strong evidence that there was no importance rating difference between the two categories. The results suggested that participants always thought that the edge-based dimensions were more important than the surfaced-based dimensions although they could classify the stimuli based on either edge-based or surface-based features.

### Discussion

The results of Experiment 1 showed that participants could simultaneously learn the categories defined by edge- and surface-based features, and importantly, there was a larger learning effect for the category defined by edge-based category than by surface-based features. Consistently, participants reported that edge-based dimensions were more important than surface-based dimensions although they could classify the stimuli based on either edge-based or surface-based features, providing convergent evidence that edge-based features matter more than surface-based features. Nonetheless, there were no differences for the two categories in the acquisition of explicit knowledge about the relation between the defined features and the category membership, indicating that the higher accuracy of the edge-based category might be due to the difference in implicit knowledge between the two categories, which means that edge-based features play a more important role than surface-based features in implicit category learning.

## Experiment 2

Based on results from Experiment 1, Experiment 2 was aimed to further investigate how the two types of information played different roles in category learning by using the ERP technique. The experimental design was identical to that in Experiment 1.

### Methods

#### Participants

Twenty-three university students (11 female, mean age = 20.42 years, *SD* = 1.36) voluntarily participated in the experiment. They were paid for their attendance. All of them reported normal or correct to normal vision. None of them had any history of neurological or psychiatric diseases. All of them were given the written informed consent. The experiment was approved by the Institutional Review Board of the Institute of Psychology, Chinese Academy of Sciences. Data from four participants were excluded from further analysis because their accuracy of both categories was below chance (0.5), and data from one participant was excluded because his accuracy was beyond 2 SDs from the mean accuracy.

#### Materials and Procedure

The stimuli and procedure were identical to Experiment 1, with exceptions that the four stimuli belonging to both categories were excluded in the training and test phases and each trial began with the fixation cross at the center for 650–950 ms at random.

#### EEG Recording and Analysis

The EEG was recorded from 64 scalp sites using Ag–AgCl electrodes in an elastic cap according to the International 10-20 system. The vertical and horizontal electrooculograms (EOGs) were recorded with two pairs of electrodes placed 1 cm above and below one eye and 1 cm lateral from the outer canthus of both eyes. The left mastoid was used as an online reference, and the algebraic average of the left and right mastoids was used as an offline re-reference. The impedance of the reference and right mastoids electrodes were maintained below 5 kΩ, and the impedance of other electrodes were maintained below 10 kΩ. The eye-movement-induced artifact was excluded by the “Ocular Artifact Reduction” module of the NeuroScan system. The EEG signals were amplified by a NeuroScan Synamps amplifier with a band pass of 0.05–100 Hz at a sampling rate of 1,000 Hz. EEG data were low-pass filtered with a cutoff frequency at 30 Hz and averaged offline for epochs of 800 ms, starting 100 ms prior to the stimulus onset in the test phase and ending 700 ms afterward. A baseline correction was performed for each epoch with respect to the 100-ms prestimulus interval. Trials with artifacts that were determined by a criterion of 50 μV were rejected offline, which amounted to only 2.9% of the trials. On average, there were 54 and 48 correct trials for the edge- and surface-based categories, respectively.

The ERPs were first averaged separately across correct and incorrect trials for the edge- and surface-based categories for each participant. In the statistical analyses of the ERP data, we focused on early components including the peak amplitudes of the posterior P1 (60–130 ms) and N1 (100–140 ms), the mean amplitudes of anterior N1 (80–130 ms) and P2 (140–180 ms), and later components including the mean amplitudes of the posterior P2 (200–240 ms) and anterior P3a (300–450 ms). On the basis of previous studies (Vogel and Luck, 2000; Chen et al., 2006; Freunberger et al., 2007; Folstein and Van Petten, 2011; Marzecová et al., 2018) and the topography of each component, a group of posterior electrodes (P3, Pz, P4, PO3, POz, PO4, O1, Oz, and O2) were selected for the posterior P1, N1, and P2; a group of anterior electrodes (F3, Fz, F4, FC3, FCz, FC4, C3, Cz, and C4) were selected for the anterior N1, P2, and P3a. To investigate whether the stimuli from the edge- and surface-based categories would produce different waveforms, the analyses were focused on the correct trials from the two categories. A 2 (category) × 9 (electrodes) within-subject ANOVA was conducted. Greenhouse–Geisser corrections were adopted when the sphericity assumption was violated (Greenhouse and Geisser, 1959).

### Results

#### Behavioral Results

##### Accuracy in the Test Phase

**Figure 4:**
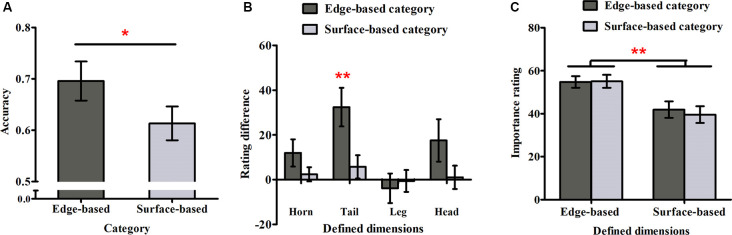
Accuracy and ratings in Experiment 2. **(A)** The accuracy for the edge- and surface-based categories in the test phase. **(B)** The probability rating difference of the defined dimensions for the edge- and the surface-based categories. **(C)** The importance rating for the defined dimensions of the edge- and surface-based categories. Error bars depict standard errors. *p < 0.05, **p < 0.01.

Figure 4A shows accuracy for each category in Experiment 2. As in Experiment 1, a one-sample *t*-test was used to examine weather participants could learn the two categories. It revealed that participants performed significantly above chance (0.50) for both categories (edge-based: *M* = 0.70, *SD* = 0.16, *t*_(17)_ = 5.16, *p* < 0.001, Cohen’s *dz* = 1.22; surface-based: *M* = 0.61, *SD* = 0.14, *t*_(17)_ = 3.44, *p* < 0.01, Cohen’s *dz =* 0.81), respectively, indicating that they learned how to classify the stimuli of the two categories incidentally. To explore the role of different features in incidental category learning, we conducted a one-tailed paired-samples *t-test*, which revealed that the accuracy for the edge-based category was significantly higher than that for the surface-based category, *t*_(17)_ = 1.86, *p* < 0.05, Cohen’s *dz* = 0.44. Thus, consistent with Experiment 1, the results confirmed that participants performed better for the category defined by edge- than by surface-based features.

##### Probability Rating

As in Experiment 1, we calculated the difference rating for each defined dimension (see Figure 4B). The one-sample *t*-test revealed that only the difference rating of tail shape for the edge-based category was significantly above zero (tail shape: *t*_(17)_ = 3.75, *p* < 0.01, Cohen’s *dz* = 0.88). The results indicated that participants were partially aware of the relation between the tail shape and the category membership only for the category defined by edge-based features. As the tail shape is one defined dimension for the edge-based category, the tail color is the corresponding defined dimension for the surface-based category. To explore whether participants could be more aware of the relation between the defined features and the categorical membership for one category than the other one, a paired-samples *t-test* was conducted on the significant difference ratings for tail. The results showed that the difference ratings for tail shape in the edge-based category was significantly higher than the difference ratings for tail color in the surface-based category (*t*_(17)_ = 3.01, *p* < 0.01, Cohen’s *dz* = 0.71), indicating that participants acquired more explicit knowledge for the edge-based category than for the surface-based category.

Furthermore, to explore whether the higher accuracy for the edge-based category was caused by the difference in explicit knowledge between the two categories, the accuracy differences between the edge- and surface-based categories was regressed on the difference between significant rating differences of tail shape and tail color. The results demonstrated that the rating difference for tail could not predict the accuracy difference in the test phase (*F*_(1, 16)_ = 1.80, *p* = 0.20), indicating that the higher accuracy for the edge-based category might be caused by the difference in implicit knowledge rather than the difference in explicit knowledge.

##### Importance Ratings

As in Experiment 1, we calculated the mean importance ratings for the four defined dimensions when participants classified the stimuli as belonging to the edge- or surface-based category separately (see Figure 4C). A 2 (dimensions: edge- vs. surface-based) × 2 (category: edge- vs. surface-based) within-subjects ANOVA revealed only a significant effect of dimensions (*F*_(1, 17)_ = 10.26, *p* < 0.01, $\eta_{\text{p}}^{2}$ = 0.38). The main effect of category and the interaction did not reach significance (*F*_(1, 17)_ = 0.57, *p* = 0.46; *F*_(1, 17)_ = 1.82, *p* = 0.20). As in Experiment 1, the Bayes factor *B* for the importance rating difference between the two categories was calculated. The mean importance rating difference between the two categories was 1.01, and the standard error of the difference was 1.35. Using the uniform range (0, 100) to represent the alternative, it yields *B* = 0.03. The results confirmed that participants always thought that the edge-based dimensions were more important than the surfaced-based dimensions although they could classify the stimuli based on either edge-based or surface-based features.

#### ERP Results

**Figure 5:**
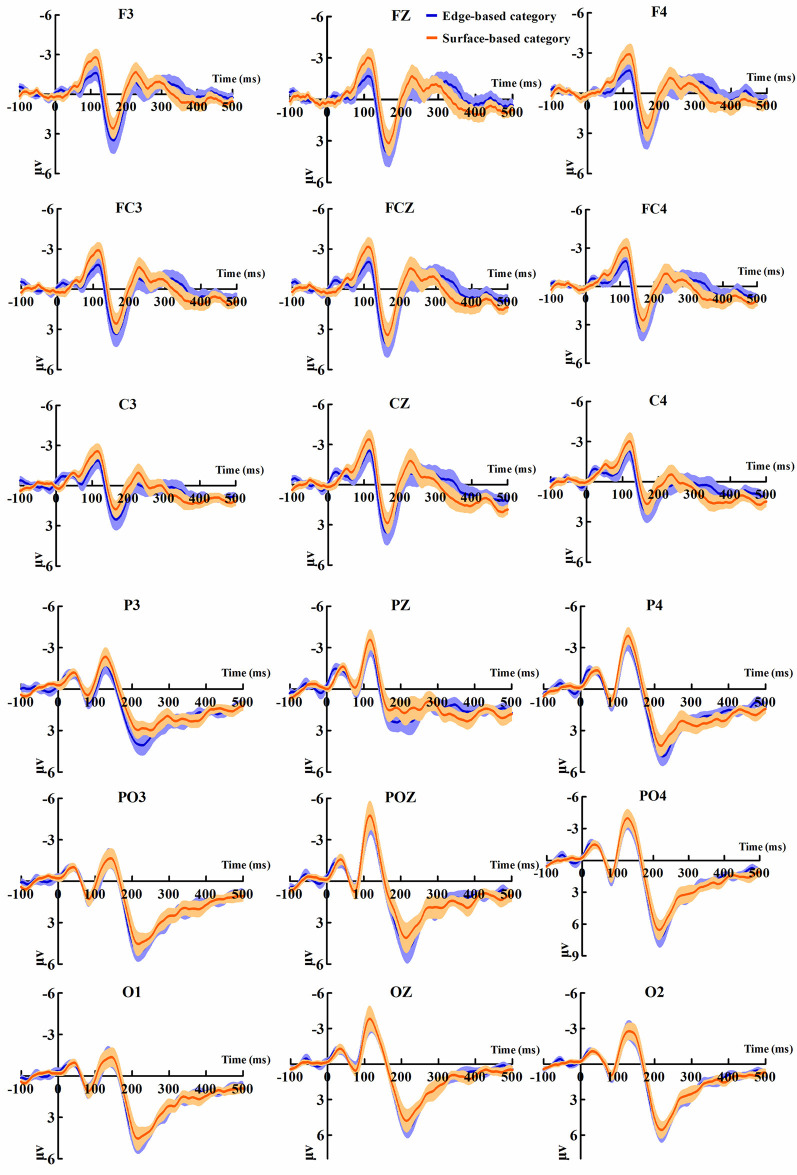
Grand-average event-related potential (ERP) waveforms of correct trials for the edge- and the surface-based categories at anterior and posterior electrodes separately. The color zone around the waveforms depicts standard errors.

**Figure 6:**
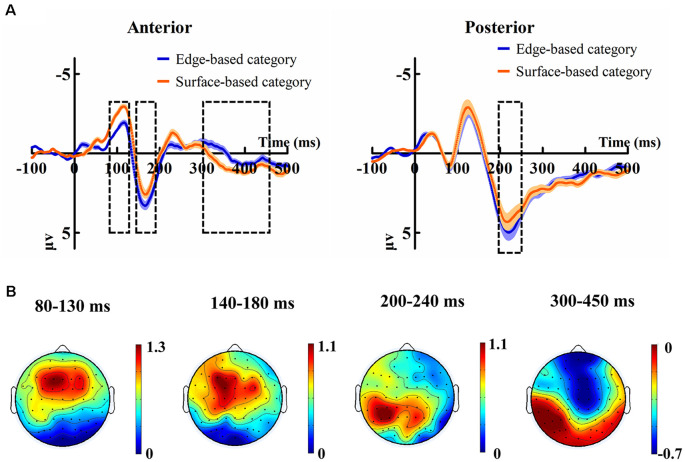
**(A)** Grand-average ERP waveforms of correct trials for the edge-based category and the surface-based category averaged across nine posterior electrodes and nine anterior electrodes, respectively. The color zone around the waveforms depicts standard errors. **(B)** The scalp topography of the anterior N1, P2, P3, and posterior P2, correct trials of the edge-based category minus correct trials of the surface-based category.

Figure 5 shows the ERP data of correct trials for the edge- and surface-based categories at each of the anterior electrodes (F3, Fz, F4, FC3, FCz, FC4, C3, Cz, and C4) and posterior electrodes (P3, PZ, P4, PO3, POZ, PO4, O1, Oz, and O2). Figure 6A shows the grand-average ERP waveforms of correct trials for the two categories averaged across nine posterior electrodes and nine anterior electrodes, respectively. Figure 6B shows the scalp topography of the anterior N1, P2, P3a, and posterior P2.

##### ERP Effects in the Early Categorization Stage

To explore the role of edge- vs. surface-based features in the early categorization stage, a 2 (category) × 9 (posterior or anterior electrodes) within-subject ANOVA was conducted on the peak amplitudes of posterior P1 and N1, as well as the mean amplitudes of anterior N1 and P2.

For the peak amplitudes of posterior P1, it revealed only a significant effect of electrodes (*F*_(4.41, 74.98)_ = 7.99, *p* < 0.001, $\eta_{\text{p}}^{2}$ = 0.32). The main effect of category (*F*_(1, 17)_ = 0.06, *p* = 0.81) and the interaction (*F*_(3.34, 56.77)_ = 1.55, *p* = 0.21) were not significant. For the peak amplitudes of posterior N1, it revealed that neither the main effects (category: *F*_(1, 17)_ = 0.86, *p* = 0.37; electrodes: *F*_(3.72, 63.26)_ = 1.70, *p* = 0.17) nor the interaction (*F*_(4.05, 68.84)_ = 1.46, *p* = 0.22) was significant.

For the mean amplitudes of anterior N1, it revealed only a significant effect of category (*F*_(1, 17)_ = 7.83, *p* < 0.05, $\eta_{\text{p}}^{2}$ = 0.32), indicating that stimuli from surface-based category elicited larger anterior N1 than those from edge-based category. The main effect of electrodes (*F*_(3.17, 53.91)_ = 1.55, *p* = 0.21) and the interaction (*F*_(3.54, 60.17)_ = 1.44, *p* = 0.24) did not reach significance.

For the mean amplitudes of anterior P2, it revealed a significant effect of category (*F*_(1, 17)_ = 5.53, *p* < 0.05, $\eta_{\text{p}}^{2}$ = 0.25), indicating that stimuli from edge-based category elicited larger anterior P2 than those from surface-based category. There was a significant effect of electrodes (*F*_(2.57, 43.69)_ = 8.96, *p* < 0.001, $\eta_{\text{p}}^{2}$ = 0.35). However, the interaction did not reach significance (*F*_(2.99, 50.78)_ = 0.32, *p* = 0.81).

##### ERP Effects in the Late Categorization Stage

To explore the role of edge- and surface-based features in the late categorization stage, a 2 (category) × 9 (posterior or anterior electrodes) within-subject ANOVA was conducted on the mean amplitudes of posterior P2 and anterior P3a.

For the mean amplitudes of posterior P2, it revealed that a significant effect of category (*F*_(1, 17)_ = 4.82, *p* < 0.05, $\eta_{\text{p}}^{2}$ = 0.22), indicating that stimuli from the edge-based category elicited larger posterior P2 than those from the surface-based category. The main effect of electrodes was significant (*F*_(3.16, 53.68)_ = 8.34, *p* < 0.001, $\eta_{\text{p}}^{2}$ = 0.33). The interaction (*F*_(2.91, 49.49)_ = 1.13, *p* = 0.35) did not reach significance.

For the mean amplitudes of anterior P3a, it revealed that the main effect of category was significant (*F*_(1, 17)_ = 5.85, *p* < 0.05, $\eta_{\text{p}}^{2}$ = 0.26), suggesting that stimuli from the surface-based category led to larger anterior P3a than those from the edge-based category. The main effect of electrodes reached significance (*F*_(2.69, 45.76)_ = 9.06, *p* < 0.001, $\eta_{\text{p}}^{2}$ = 0.35). The interaction did not reach significance (*F*_(3.16, 53.79)_ = 1.57, *p* = 0.21).

##### The Relation Between Behavioral Data and ERP Data

To examine the relation between ERPs and behavioral performance, we calculated the accuracy difference between the edge- and the surface-based categories and the mean amplitude differences for anterior N1, P2, P3, and posterior P2. Then, the accuracy differences between the two categories were regressed on the mean amplitude differences for anterior N1, P2, P3, and posterior P2. The stepwise regression showed that only the mean amplitude differences of anterior P3a could significantly predict the accuracy differences between the edge- and surface-based category in the test phase (*F*_(1, 17)_ = 4.82, *p* < 0.05) with an adjusted *R^2^* of 0.18.

### Discussion

The behavioral results of Experiment 2 replicated the main findings in Experiment 1, indicating that participants learned better for the edge-based category than for the surface-based category, confirming that edge-based features play a more crucial role than surface-based features in incidental category learning. Importantly, the ERP results revealed that there were larger anterior N1 but smaller anterior P2 for the surface-based category than for the edge-based category, indicating that stimuli from the surface-based category might attract more attention but less feature analysis was done for them compared with those from the edge-based category at the early categorization stage. Moreover, there were smaller posterior P2 but larger anterior P3a for the surface-based category than for the edge-based category, suggesting that edge-based information plays more important roles in evaluating information relevance in making a decision at the late categorization stage.

## General Discussion

The behavioral results showed that knowledge for both edge- and surface-based categories could be simultaneously acquired in incidental category learning, and importantly, there was a larger learning effect for the edge-based category than for the surface-based category. Consistently, participants reported that edge-based dimensions were more important than surface-based dimensions although they could classify the stimuli based on either edge-based category or surface-based features. The ERP results revealed that the stimuli from the edge-based category elicited larger anterior P2 and posterior P2 than those from the surface-based category, while stimuli from the surface-based category elicited larger anterior N1 and P3a than those from the surface-based category. The results provided new behavioral and ERP evidence that edge- and surface-based features play different roles in incidental category learning. That is, although surface-based information might attract more attention during feature detection, edge-based information plays more important roles in evaluating the relevance of information in making a decision in categorization.

Participants were asked to observe each cartoon animal and listen to the sound carefully and then rate how likeable they were in the training phase. They were not asked to learn the category directly, and no trial-by-trial feedback was provided in both the training phase and the test phase. This guaranteed that the learning process occurred incidentally. Under these circumstances, participants performed above chance for both categories, indicating that they could incidentally combine the sound and the defined features to form the category knowledge and use it in the test phase. Otherwise, the accuracy for one category would be at chance level. Importantly, there was a larger learning effect for the edge- than for the surface-based category, and the larger learning effect was caused by the difference in implicit knowledge between the two categories rather than the difference in explicit knowledge, confirming that edge-based features play a more crucial role than surface-based features in implicit category learning.

The edge-based theory, such as Biederman’s recognition-by-components model, posits that objects are recognized based on their shape properties (Biederman, 1987; Biederman and Ju, 1988). Consistently, several studies have further demonstrated that edge-based information is a principal discriminative cue and its influence emerges earlier than texture and color (Elder and Velisavljević, 2009; Rokszin et al., 2015). For example, when extracting an average orientation from a set of objects, performance has been found to be better when the orientation is carried by the boundary features of the objects, relative to when it is carried by the surface features of the objects (Choo et al., 2012). Thus, the behavioral results of our two experiments provide new evidence for the edge-based theory and extend the application of this theory from object recognition to category learning.

Our ERP results revealed that the amplitude of anterior N1 was larger for the surface- than for the edge-based category, indicating that the stimuli from the surface-based category might attract more attention compared with the stimuli from the edge-based category. As stimuli from both edge- and surface-based categories include five edge-based features and five surface-based features, there should be no difference on feature saliency between the two categories. That is, this attention effect might not be due to a stimulus-driven attentional capture (e.g., Cave, 1999; Turatto and Galfano, 2000; Müller et al., 2009). This is consistent with the finding that the posterior P1 and N1 are not significantly different between stimuli from the two categories. Thus, the attention effect might be modulated by a top–down mechanism (Connor et al., 2004; Theeuwes, 2010). The information of the stimulus can be rapidly projected from early visual areas directly to the prefrontal cortex resulting in a coarse representation, which is subsequently used to activate predictions about the most likely interpretations of the stimulus (Bar et al., 2006; Schettino et al., 2011). If the category representation consists of mainly edge-based features, the coarse representation of stimuli for the edge-based category can be formed more easily than that for the surface-based category. Therefore, more top–down attention is needed for stimuli from the surface-based category than for the edge-based category, as reflected by a larger anterior N1 for the surface-based category than for the edge-based category. These results are also consistent with a previous study during which participants needed to decide if the probe stimulus share the same category membership of the previous two stimuli (Bigman and Pratt, 2004), and which revealed that a larger N1 could be recorded in response to the first stimulus when the knowledge of the target feature was unknown and the attention was needed for all features during processing of it compared with the second stimuli and the probe.

However, the ERP results revealed that the amplitude of anterior P2 was larger for the edge-based category than for the surface-based category. Relative to the condition under which participants are instructed to discriminate between old and new objects, the enhanced anterior P2 has been found in the condition under which they need to decide additionally whether old objects are larger or smaller since the more extensive evaluation of specific perceptual attributes is engaged (Ranganath and Paller, 2000). It has also been found for word targets from which target visual features can be more efficiently extracted when they are congruent with the context (Federmeier et al., 2005). These studies suggest that the anterior P2 reflects the detection of visual features with feature-based attention (Luck and Hillyard, 1994; Dunn et al., 1998; Luck, 2012). Because the anterior P2 is larger for the edge-based category than for the surface-based category, the anterior P2 component might reflect that the edge-based features could be detected and analyzed more efficiently than the surface-based features.

From the view of bottom–up visual processing, after processing the presented object, the perceptual information is matched to the representation in memory to make decisions (Ungerleider and Bell, 2011; Taminato et al., 2014). It has been found that the older adults with working memory encoding decrements have lower posterior P2 amplitude than young adults in a modified Sternberg recognition task (Finnigan et al., 2011), and correct trials elicit larger posterior P2 than incorrect trials in a digit span backward task (Lefebvre et al., 2005). The results suggest that the posterior P2 reflects the cognitive matching process. Consistent with this, our research shows that stimuli from edge-based category elicit larger posterior P2 than that from surface-based category, suggesting that edge-based information from the current stimulus can be better evaluated and compared with the stored inner categorical representation.

The P3a component has been proposed as an index of stimulus categorization (Johnson and Donchin, 1980; Dien et al., 2004). Folstein and Van Petten have separated that categorization into a dual system: a relatively fast process if the category is defined by a single- or two-feature conjunctions as indexed by the posterior P3b, and a slower process engaged when the number of relevant features exceeds two as indexed by the P3a, which are late positive potentials at frontal scalp sites (Folstein and Van Petten, 2004, 2011). As the category in the present study is defined by four features and the surface- and edge-based categories differ in the P3a, the results provide supportive evidence for the two dual category systems (Folstein and Van Petten, 2004, 2011). The larger P3a for the surface-based category than for the edge-based category is also consistent with previous studies showing that the anterior P3a might reflect a mixture of category selectivity and categorization uncertainty with enhanced responses to uncertain stimuli (Scholl et al., 2014). Because the difference in the P3a amplitudes between the two categories could predict the accuracy difference, the relatively poor accuracy for the surface-based category might be due to the difficulty in evaluating the surface-based features with internal representations.

In summary, the current study suggests that the edge-based features play a more important role than surface-based features. Furthermore, although the surface-based features attract more attention at the early stage of classification, it is the edge-based features that play a more crucial role in retrieving internal representations and evaluating the relevant information in decision making at the late stage of classification.

## Data Availability Statement

The raw data supporting the conclusions of this article will be made available by the authors, without undue reservation, to any qualified researcher.

## Ethics Statement

The studies involving human participants were reviewed and approved by the Institutional Review Board of the Institute of Psychology, Chinese Academy of Sciences. The patients/participants provided their written informed consent to participate in this study.

## Author Contributions

XZ and QF designed the experiment. XZ performed the experiment and analyzed the collected data. XZ, QF, and MR wrote and revised the manuscript.

## Conflict of Interest

The authors declare that the research was conducted in the absence of any commercial or financial relationships that could be construed as a potential conflict of interest.
